# SUBMIT: Systemic therapy with or without up front surgery of the primary tumor in breast cancer patients with distant metastases at initial presentation

**DOI:** 10.1186/1471-2482-12-5

**Published:** 2012-04-02

**Authors:** Jetske Ruiterkamp, Adri C Voogd, Vivianne CG Tjan-Heijnen, Koop Bosscha, Yvette M van der Linden, Emiel JTh Rutgers, Epie Boven, Maurice JC van der Sangen, Miranda F Ernst

**Affiliations:** 1 Department of Surgery, Máxima Medical Center, Veldhoven, The Netherlands; 2 Department of Epidemiology, GROW--School for Oncology and Developmental Biology, Maastricht University Medical Centre, Maastricht, The Netherlands; 3 Division of Medical Oncology, Department Internal Medicine, GROW--School for Oncology and Developmental Biology, Maastricht University Medical Centre, Maastricht, The Netherlands; 4 Department of Surgery, Jeroen Bosch Hospital, 's-Hertogenbosch, The Netherlands; 5 Radiotherapeutic Institute Friesland, Leeuwarden, The Netherlands; 6 Department of Surgical oncology, Dutch Cancer Institute--Antoni van Leeuwenhoek Hospital, Amsterdam, The Netherlands; 7 Department of Medical Oncology, VU University Medical Centre, Amsterdam, The Netherlands; 8 Department of Radiotherapy, Catharina Hospital, Eindhoven, The Netherlands; 9 Department of Surgery, Jeroen Bosch Hospital, Postbus 90153, 5200 ME 's-Hertogenbosch, The Netherlands

**Keywords:** Primary metastatic breast cancer, surgery, randomised controlled trial

## Abstract

**Background:**

Five percent of all patients with breast cancer have distant metastatic disease at initial presentation. Because metastatic breast cancer is considered to be an incurable disease, it is generally treated with a palliative intent. Recent non-randomized studies have demonstrated that (complete) resection of the primary tumor is associated with a significant improvement of the survival of patients with primary metastatic breast cancer. However, other studies have suggested that the claimed survival benefit by surgery may be caused by selection bias. Therefore, a randomized controlled trial will be performed to assess whether breast surgery in patients with primary distant metastatic breast cancer will improve the prognosis.

**Design:**

Randomization will take place after the diagnosis of primary distant metastatic breast cancer. Patients will either be randomized to up front surgery of the breast tumor followed by systemic therapy or to systemic therapy, followed by delayed local treatment of the breast tumor if clinically indicated.

Patients with primary distant metastatic breast cancer, with no prior treatment of the breast cancer, who are 18 years or older and fit enough to undergo surgery and systemic therapy are eligible. Important exclusion criteria are: prior invasive breast cancer, surgical treatment or radiotherapy of this breast tumor before randomization, irresectable T4 tumor and synchronous bilateral breast cancer. The primary endpoint is 2-year survival. Quality of life and local tumor control are among the secondary endpoints.

Based on the results of prior research it was calculated that 258 patients are needed in each treatment arm, assuming a power of 80%. Total accrual time is expected to take 60 months. An interim analysis will be performed to assess any clinically significant safety concerns and to determine whether there is evidence that up front surgery is clinically or statistically inferior to systemic therapy with respect to the primary endpoint.

**Discussion:**

The SUBMIT study is a randomized controlled trial that will provide evidence on whether or not surgery of the primary tumor in breast cancer patients with metastatic disease at initial presentation results in an improved survival.

**Trial registration:**

NCT01392586.

## Background

In most western countries, around five percent of all patients with breast cancer have distant metastatic disease at initial presentation [1,2]. This accounts for 500 newly diagnosed patients each year in the Netherlands [1]. Because metastatic breast cancer is considered to be an incurable disease, the aim of the treatment for these patients is to provide palliation amongst others with systemic therapy. Usually, breast surgery is restricted to those patients in whom the breast tumor is symptomatic. The rationale behind this strategy is based on the fact that once distant metastases have occurred, (aggressive) local therapy provides no survival advantage.

**Figure 1:**
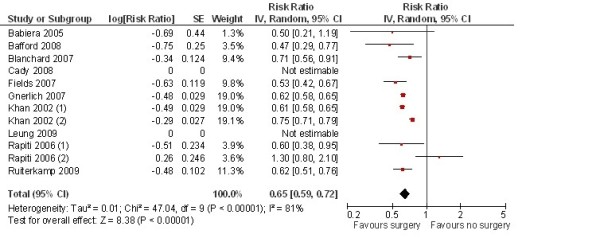
**Pooled analyses of hazard ratios for overall mortality for surgery versus no surgery for patients with stage IV breast cancer **[**14**]. (1): patients with free surgical margins; (2): patients with positive surgical margins.

Research on the effect of systemic therapy in women with metastatic disease has demonstrated that their prognosis has improved significantly during the last 10 to 15 years, primarily due to increased efficacy of chemotherapy and the introduction of targeted treatments [2-4]. Recent retrospective studies have demonstrated that resection of the breast tumor in patients with primary metastatic breast cancer is associated with a significant improvement of the prognosis (Table 1) [5-12]. The hazard ratios (HR) for overall survival in these studies ranged from 0,50 to 0,71 in favor of surgery of the breast tumor. Furthermore, in studies taking surgical resection margins into account, better survival was observed in patients with a primary breast lesion that had been removed with free surgical margins [5,7,13]. In the review of Ruiterkamp et al. a pooled HR of 0.65 (95% confidence interval 0.59-0.72) was calculated for overall survival for surgery versus no surgery, in favor of surgery (Figure 1) [14]. Results of a stratified analysis, done by Rapiti et al. suggest a greater effect for surgery among women with only bone metastases at diagnosis [7].

**Table 1:** Results of retrospective studies

Author	Year	Nr of patients	Surgery (%)	HR	95% CI	Median survival (months)			
						
						Surgery			No surgery
							
						Not specified	Lumpec	Mastec	
Khan [5]	2002	16023	57	0.61	0.58-0.65	-	27	32	19

Babiera [6]	2006	224	37	0.50	0.21-1.19	-			-

Rapiti [7]	2006	300	42	0.60	0.4-1.0	-^1^			-

Fields [8]	2007	409	46	0.53	0.42-0.67	32			15

Gnerlich [9]	2007	9734	47	0.63	0.60-0.66	36			21

Blanchard [10]	2008	395	61	0.71	0.56-0.91	27			17

Cady^2 ^[15]	2008	622	38	-	-	-			-

Bafford [16]	2009	147	41	0.47	-	42			28

Ruiterkamp [11]	2009	728	40	0.62	0.51-0.76	31			14

Leung [17]	2009	157	33	-	-	25			13

Neuman [12]	2010	186	37	0.71	0.47-1.06	40			33

Dominici^2 ^[18]	2011	290	23	0.94	0.83-1.08	42			41

HR: Hazard ratio; 95% CI: 95% confidence interval

^1 ^Rapiti: 5-year specific survival; 27% for surgery with negative margins, 16% for surgery with positive margins, 12% for surgery with unknown margins and 12% for no surgery

^2 ^Case-matched analysis

In all these aforementioned studies, the decision to perform surgery could have been influenced by favorable prognostic factors, such as younger age, the presence of only one metastatic site or a good response to systemic therapy. To rule out the impact of potential confounders, most studies adjusted for age, tumor size, number and sites of metastases and hormone receptor status. In addition, some studies also adjusted for comorbidity or surgical margins.

More recent studies have investigated the role of additional confounding factors, such as timing of surgery, selection bias and coding errors, and came to the conclusion that the survival benefit seen in patients who were treated with a surgical resection of the primary tumor was not so strong or may have been explained by these confounders [15-18]. For example, in the study of Bafford et al., the benefit of surgery seemed confined to patients operated upon before diagnosis of metastatic disease and there was no survival advantage in patients who received an operation of the breast tumor after the diagnosis of the metastatic disease had taken place. This phenomenon was referred to as stage migration bias [16,18]. In a study by Leung et al., the benefit from surgery disappeared in the multivariate analysis when taking into account the use of chemotherapy [17]. Finally, in a study by Cady et al. coding errors in the retrospectively collected dataset were found to explain part of survival advantage [15].

Given the nature of retrospective analysis, it is not possible to provide a definite answer to the question whether surgical therapy of the breast tumor indeed affects overall survival. Therefore, in the Netherlands a randomized controlled trial (RCT) has been initiated and will start recruiting in the second half of 2011. This study is called, the SUBMIT trial, an acronym for '**S**ystemic therapy with or without **U**p front surgery of the primary tumor in **B**reast cancer patients with distant **M**etastases at **I**nitial presen**T**ation'. In the current paper we will present the design of this trial.

### Design

**Figure 2:**
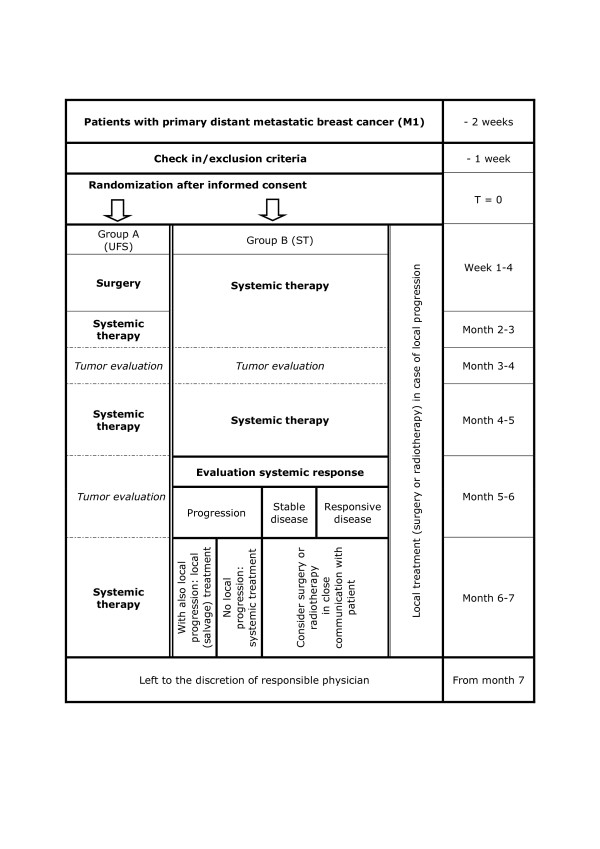
**Study design**.

The aim of the SUBMIT study is to investigate the effect of surgery of the primary tumor in breast cancer patients with distant metastatic disease at initial diagnosis. After diagnosis of primary distant metastatic breast cancer, patients will be randomly allocated in two groups: A. Up front breast surgery followed by systemic therapy; B. Systemic therapy potentially followed by delayed local treatment of the breast tumor (Figure 2).

### Patient selection criteria

The eligibility criteria for the study are:

- primary distant metastatic breast cancer (M1); metastases diagnosed

- within one month after the diagnosis of the breast cancer

- an anticipated survival of at least 6 months;

- a histologically proven diagnosis of the breast tumor;

- a known hormonal and HER2Neu status;

- TNM classification: T1-T3, resectable T4 status and N0-N3;

- performance status and comorbidity should allow surgery and/or systemic therapy;

- age ≥ 18 years;

- written informed consent.

Exclusion criteria are:

- a primary invasive breast cancer in the medical history;

- another malignancy within the last 10 years, besides basal cell carcinoma of the skin or early stage cervical cancer;

- surgical treatment and/or radiotherapy of the breast tumor before randomization;

- irresectable T4 breast tumor;

- synchronous bilateral breast cancer.

### Randomization

Centralized randomization will take place immediately after the diagnosis of primary distant metastatic breast cancer, using a computer-generated randomization list. Patients either randomize for up front surgery of the breast tumor followed by systemic therapy or for systemic therapy possibly followed by local treatment of the breast tumor if clinically indicated.

The randomization will be balanced by minimization, according to the minimization algorithm of Pocock and Simon [19], for: centre, age (18-49, 50-69, ≥ 70 years), dominant location of distant metastases (bone, soft tissue (lymph nodes and subcutaneous metastases) and visceral metastases), hormonal receptor status (ER and/or PR positive or both negative) and HER2Neu status (overexpression yes or no).

Randomization will be performed centrally, using a website with electronic data-entry to check treatment eligibility.

### Hypotheses

The following three hypotheses will be tested:

1. Up front breast surgery in patients with primary distant metastatic breast cancer will result in a significant improvement of the 2-year survival compared to the survival achieved with palliative systemic therapy followed by delayed local treatment or systemic therapy alone.

2. Local tumor control in these patients will be superior in case of up front breast surgery compared to patients who receive systemic treatment with delayed local therapy or systemic therapy alone.

3. Better local control, by the use of up front breast surgery, results in a better quality of life in patients with Stage IV breast cancer, compared to patients who receive systemic treatment with delayed local therapy or systemic therapy alone.

### Ethical approval

The study is approved by the one of the Dutch Medical Ethics Committees (METOPP; Medisch-Ethische Toetsing Onderzoek Patiënten en Proefpersonen, Tilburg). The project number is NL30331.028.11. The METOPP is an Ethical Review Committee according to the Dutch Medical Research Involving Human Subjects Act (WMO: Wet Medisch-Wetenschappelijk Onderzoek met Mensen). This study will be carried out in compliance with the Helsinki Declaration [20].

### Settings and location

The study is a multicenter randomized controlled trial, which will be conducted in the Netherlands. Both academic and regional hospitals are allowed to participate, and already 25 centers are willing to participate.

### Treatment options

During the study patients can be treated with surgery of the primary tumor (depending on randomization), systemic therapy and/or locoregional radiotherapy. The different treatment modalities are described below.

#### Surgery of the primary tumor and axillary lymph node dissection

Patients who are randomized to up front surgery of the breast tumor will receive a lumpectomy or mastectomy depending on patient and tumor characteristics. Both types of surgery may be conducted as long as the intention is a complete resection of the primary tumor, which is defined as having succeeded in obtaining free resection margins for the invasive component. In case of the presence of involved margins (more than focally)there are three options:

- to perform a re-excision or mastectomy (preferred options in up front surgery group);

- to treat the patient with locoregional radiotherapy;

- to accept that a non-radical resection has been performed.

What to decide belongs to the responsibility of the treating physician; he or she is not bound by restrictions in the protocol.

Also the choice to perform an axillary lymph node dissection is left to the discretion of the treating physician, but is highly recommended if palpable and/or tumor positive lymph nodes in the axillary region are present.

If patients are randomized for systemic therapy, they may still be candidates for surgery of the primary tumor. In case of local progression breast surgery is allowed at any time to gain local control, but preferentially not within the initial 5-6 months of first-line systemic therapy. Surgery for this group is primary indicated when the treating physician beliefs the tumor may cause wound problems in near future, despite use of systemic therapy.

#### Locoregional radiotherapy

There are two situations where radiotherapy to the breast or chest wall should be considered. First after an irradical lumpectomy or mastectomy. Irradicality in this trial is defined as involved margins for invasive breast cancer. The second situation is in case of local progression in patients in group B where a non invasive local therapy with radiotherapy is a treatment option instead of surgery. In case of clinically positive axillary lymph nodes, radiotherapy may be a viable treatment option instead of surgery as well. In both situations a hypofractionated regimen is mandatory without too much delay in systemic therapy, if applicable. Of note,--if considered indicated--radiotherapy can be postponed after first-line chemotherapy has been delivered. Concurrent radiotherapy and chemotherapy is not allowed because of expected excessive toxicity.

Radiotherapy may also be indicated for distant metastases, such als painful bone metastases or brain metastases.

#### Systemic therapy

Patients will be treated with systemic therapy according to the guidelines from the NAtional Breast cancer Organization of the Netherlands (NABON) for treatment of breast cancer. The choice of initial chemotherapy, immunotherapy and endocrine therapy depends--among others--on ER and PR and HER2Neu status, dominant site of distant metastases, age, performance status and comorbidity; but may also depend on the chance to reach a complete remission for example in minimal metastatic disease. In patients with a hormone receptor positive tumor, hormonal treatment in indicated. In case of rapid progression, chemotherapy is the treatment of choice. In HER2Neu positive disease and the use of an aromatase inhibitor, it is possible to add HER2Neu targeted therapy. Chemotherapy is offered to patients with hormone receptor negative status, with extensive and fast growing visceral metastases and with severe cytopenia. For this study we advice to use at least an anthracycline, a taxane and capecitabine for the first 2-3 lines of treatment. The order is left to the discretion of the treating physician, if necessary in combination with targeted therapy, such as bevacizumab or HER2Neu targeted therapy, according to local practice. Patients with HER2Neu overexpression, can be treated with a combination of trastuzumab and taxanes as first-line chemotherapy. At first line treatment one may also choose for anthracycline containing chemotherapy, without HER2Neu targeted therapy. During the following lines of chemotherapy, continuing HER2Neu blocking with trastuzumab or lapatinib is advised.

### Primary and secondary outcome measures

The primary endpoint is the two-year survival. This is defined as the percentage of patients who survive two years after randomization. Secondary endpoints are quality of life, overall survival, number of unplanned local therapies, i.e. surgery or radiotherapy, number of axillary lymph node dissections or axillary radiotherapy, determination of pathological resection margin (margin status) in patients treated by surgery of the primary tumor and type of chemotherapy, immunotherapy and endocrine therapy and number of regimens of systemic therapy during the first 2 years.

### Statistics

#### Sample size calculation

In a previous, retrospective, study performed in the south of the Netherlands, the median survival of patients with stage IV breast cancer who had surgery was 31 months, as compared to 14 months for patients who did not have surgery (*P *< 0.0001) [11]. In a multivariable Cox regression analysis, adjusting for age, period of diagnosis, T-classification, number of metastatic sites, co-morbidity, use of locoregional radiotherapy and use of systemic therapy, the HR of breast surgery for overall mortality in this study was 0.62 (95% confidence interval (CI) 0.51-0.76). In a recently published meta-analysis, including the results of 9 retrospective studies, the pooled HR for overall mortality was 0.65 (95% CI 0.59-0.72) [14].

We are planning a randomized controlled trial with an equal number of patients in both treatment arms, an accrual interval of 60 months, and additional follow-up after the accrual interval of 18 months. We assume that (as a result of more effective systemic treatments) the median survival time of the patients without surgery has improved to 18 months since 2004, which was the last period of diagnosis included in the retrospective study based on the data from the Eindhoven Cancer Registry [1]. If the true hazard ratio (relative risk) of the patients with upfront surgery relative to the patients with immediate start of systemic therapy is 0.76 (the upper boundary of the 95% CI of the retrospective study, performed in the south of the Netherlands by Ruiterkamp et al. [11]), we will need to study 248 patients with up front surgery and 248 without up front surgery to be able to reject the null hypothesis that the survival curves are equal with a probability (power) of 0.80. The Type I error probability associated with this test of this null hypothesis is 0.05. An additional number of 20 patients will be included to account for loss to follow-up and/or exclusion after randomization because of violation of the eligibility criteria; so total accrual consists of 516 patients.

#### Data analysis

Investigators will enter the information required by the protocol into the Case Report Forms (CRFs). The data from all centers will be pooled and summarized with respect to demographic and baseline characteristics and efficacy and safety observations. Data will be presented for the complete intent-to-treat population. The primary endpoint will be analyzed in a Cox regression model, with the minimization factors as covariables.

#### Interim analysis

An interim analysis will be performed after 50% (258 patients) of the total required number of patients has been included. The purpose of this interim review is to assess any clinically significant safety concerns and to determine whether there is evidence that up front surgery (treatment A) is clinically or statistically inferior to immediate systemic therapy without up front surgery (treatment B), with respect to the primary endpoint. To control the overall type I error when performing the interim analysis, the Peto approach will be used to ensure an overall Type I error of 5% [21,22]. A one-sided significance level of 0.001 will be used at the interim analysis.

## Discussion

Recent studies on surgery of the breast tumor in patients with primary distant metastatic disease are inconclusive regarding the effect of surgery on overall survival. Most indicate that surgical treatment is associated with a significantly improved overall survival [5-12], but some state that this benefit is caused by confounding, induced by the retrospective study designs [15-18]. In order to provide a definite answer with respect to the role of surgery in primary metastatic breast cancer, a prospective randomized controlled trial, the SUBMIT study, is about to be initiated within The Netherlands. If surgery is shown to be associated with improved survival, it would also be interesting to know more about the biological mechanisms which underly the effect. Therefore, research on circulating tumor or endothelial cells in blood, the immune response and the angiogenic potential of metastases will be considered within the trial. A grant application for a side-study, in which circulating tumor cells (CTCs) will be enumerated and characterized for HER2Neu expression and estrogen receptor status, has already been submitted. This side-study would enable us to address the hypothesis that among patients with a HER2Neu negative primary tumor those with HER2Neu positive CTCs have a worse outcome to standard systemic treatment compared to those with HER2Neu negative CTCs. Additionally, the impact of primary tumor resection on CTC numbers will be analyzed.

## Competing interests

The authors declare that they have no competing interests.

## Authors' contributions

All authors made substantive intellectual contributions to the study protocol. They have been involved in drafting the manuscript and read and approved the final version of the manuscript. Conception and design; JR, ME, KB, AV, VT, ER, EB. Collection and assembly of data; ME, AV, VT, YL, MS. Manuscript writing; JR, ME, AV, YL, VT, MS. Final approval of manuscript; JR, ME, KB, AV, YL, VT, ER, EB, MS.

## Funding

A grant of Sanofi Aventis was used for the initiation of this study.

Funding for the study is provided by the Dutch Cancer Society (Stichting Koningin Wilhelmina Fonds (KWF) voor de Nederlandse Kankerbestrijding). The project number is JBZ 2011-4995. This grant covers the costs for data management of the clinical study and some additional expenses.

## Pre-publication history

The pre-publication history for this paper can be accessed here:

http://www.biomedcentral.com/1471-2482/12/5/prepub
